# Supplementary figures and images for: Genetic mapping of fitness determinants across the malaria parasite Plasmodium falciparum life cycle
Source: PLoS Genet. 2019 Oct 14;15(10):e1008453. doi: 10.1371/journal.pgen.1008453 (PMC6821138; doi:10.1371/journal.pgen.1008453)

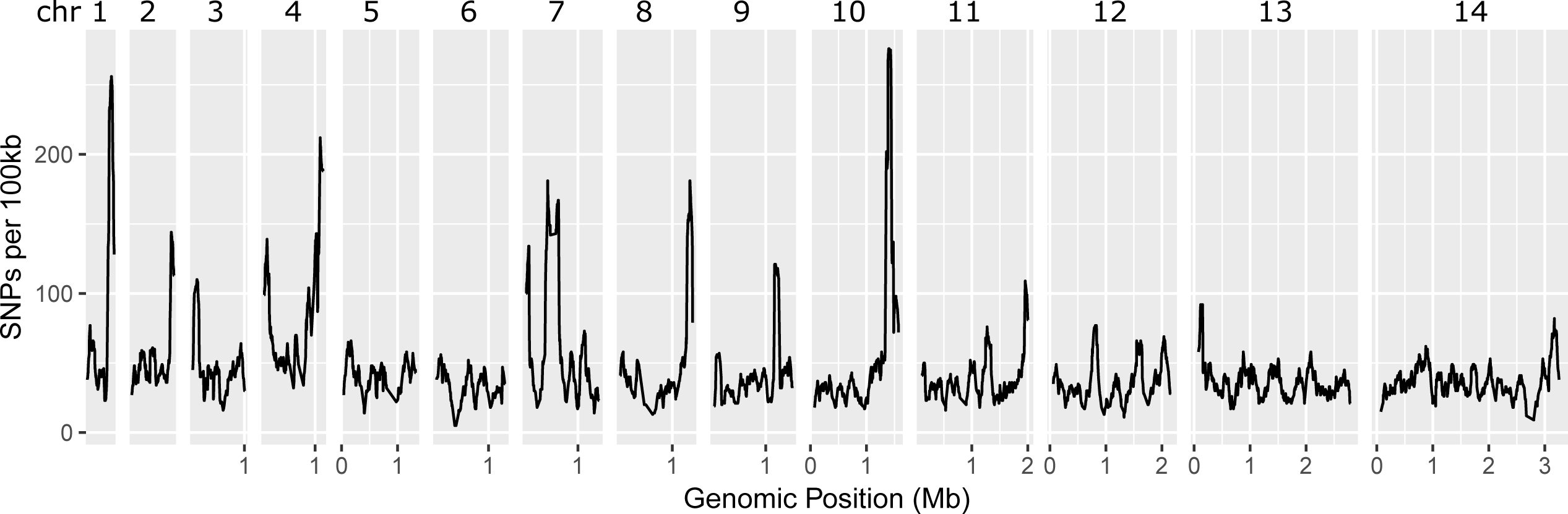

Supplement: S1 Fig — NHP1337 and MKK2835 differ from the core genome sequence of 3D7 (PlasmoDB, release32) by 13,762 and 13,710 SNPs, respectively. (TIF) [file pgen.1008453.s001.tif]

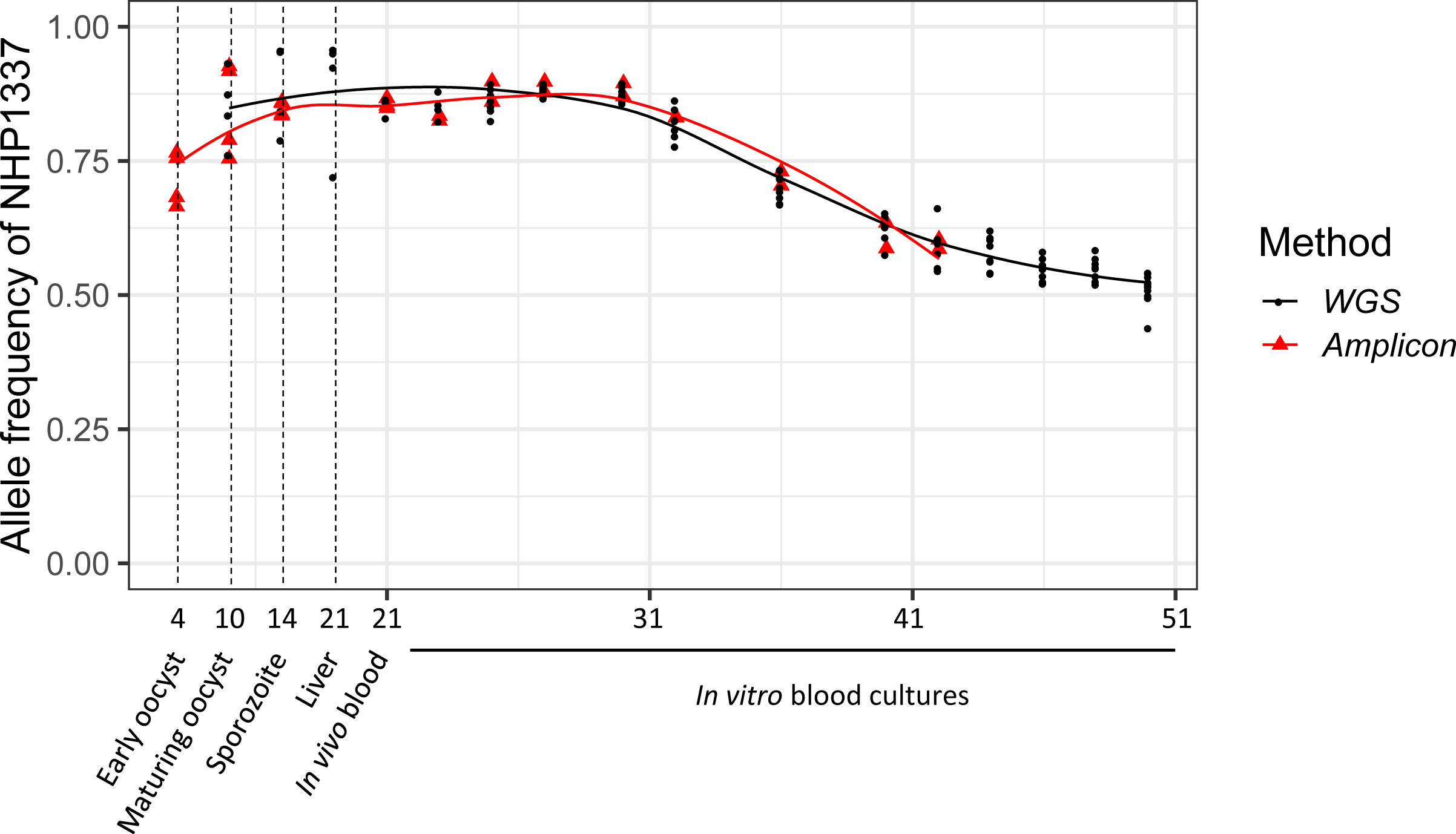

Supplement: S2 Fig — Mitochondrial allele frequencies estimated by amplicon sequencing (red) and whole-genome sequencing (black). (TIF) [file pgen.1008453.s002.tif]

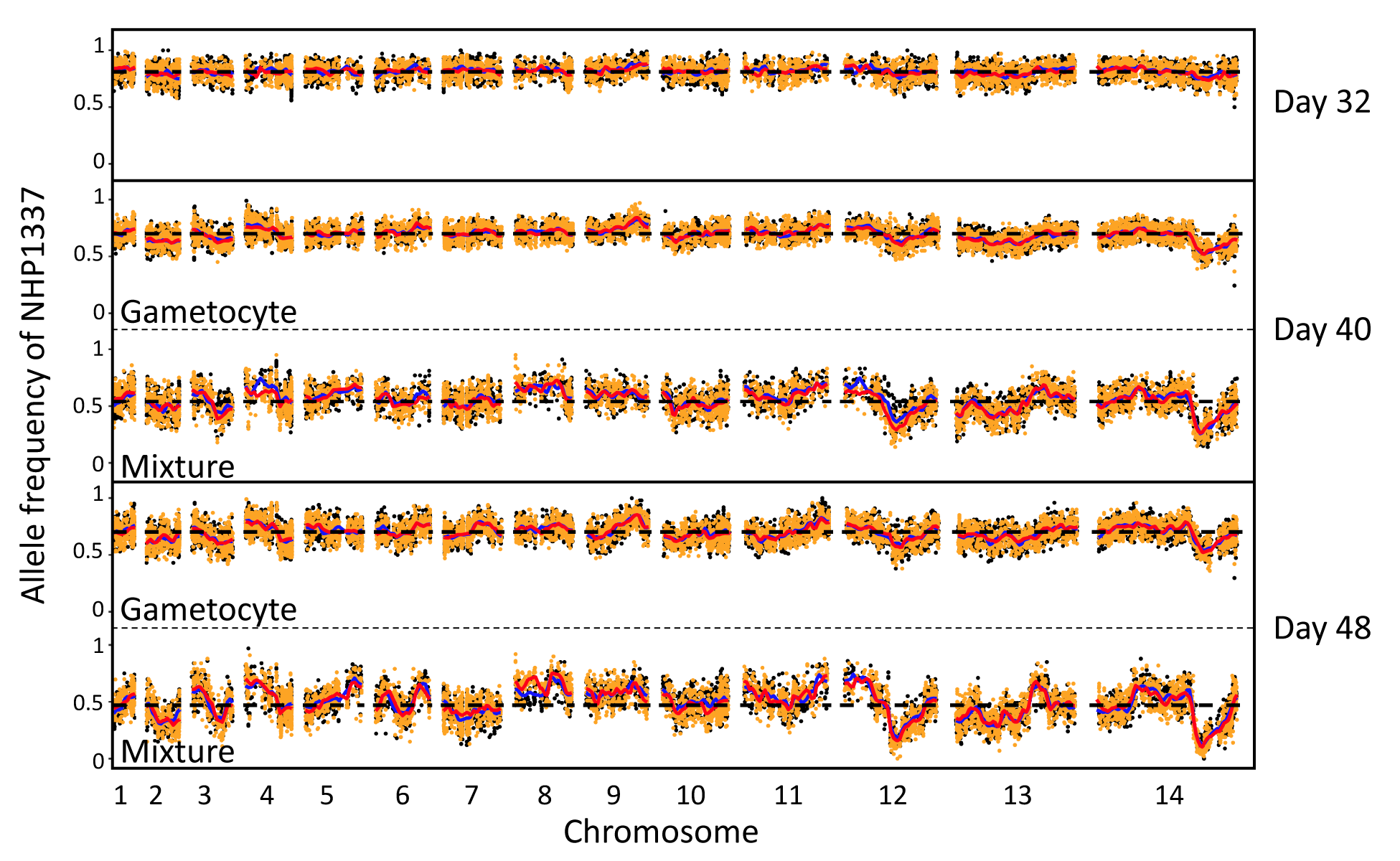

Supplement: S3 Fig — The enrichment was initiated at day 32. We collected samples for sequencing 8 days (day 40) and 16 days (day 48) later. We compared allele frequencies between gametocyte enrichment cultures (marked as “Gametocyte”) and normal in vitro cultures (marked as “Mixture”) which contained both asexual and sexual parasites. (TIF) [file pgen.1008453.s003.tif]

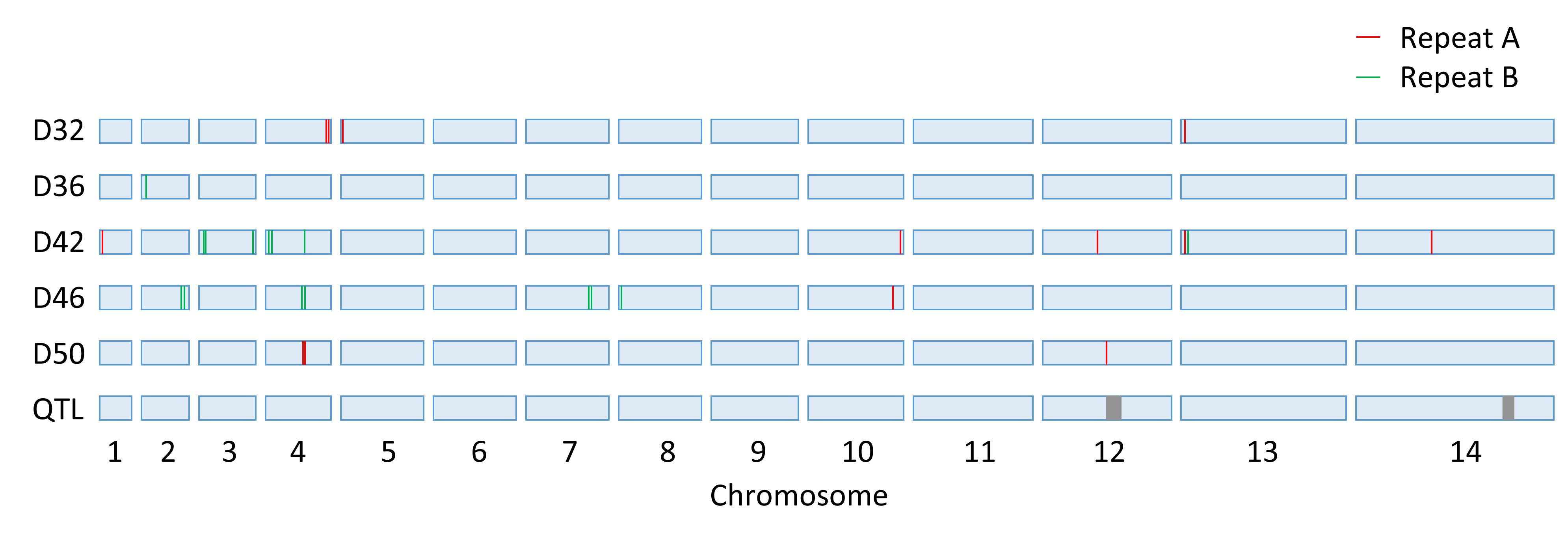

Supplement: S5 Fig — Location of possible allele frequency jumps (see S4 Table for details) detected are marked by vertical lines; Repeat A and B represent results from two parallel in vitro blood cultures; QTL regions located at chr 12 and 14 are marked in grey. The chr 12 QTL has an allele frequency jump detected in the day50 sample in one of the two replicates. No jumps we detected close to the chr 14 in either replicate in the temporal samples examined. (TIF) [file pgen.1008453.s005.tif]

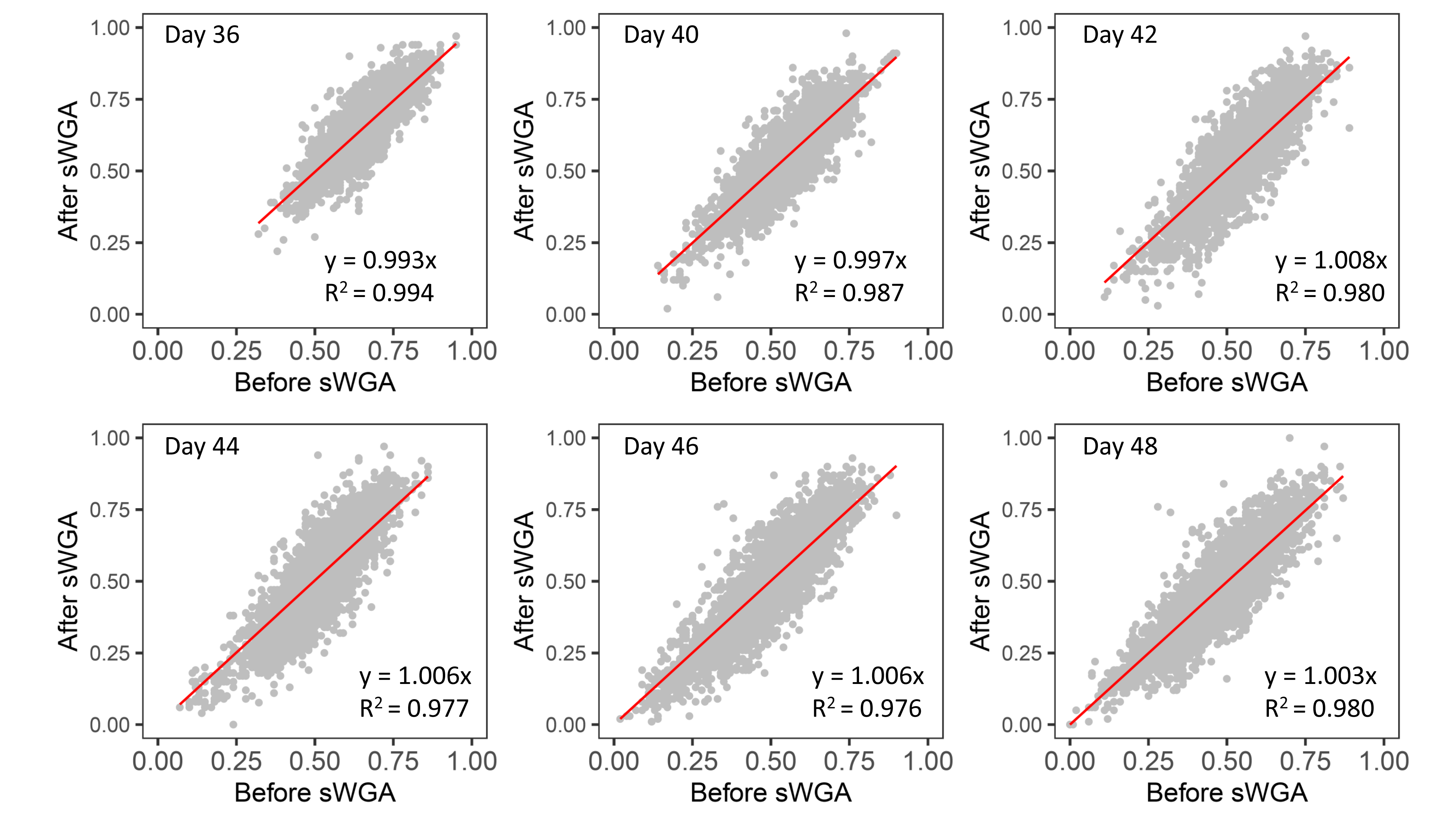

Supplement: S6 Fig — (TIF) [file pgen.1008453.s006.tif]

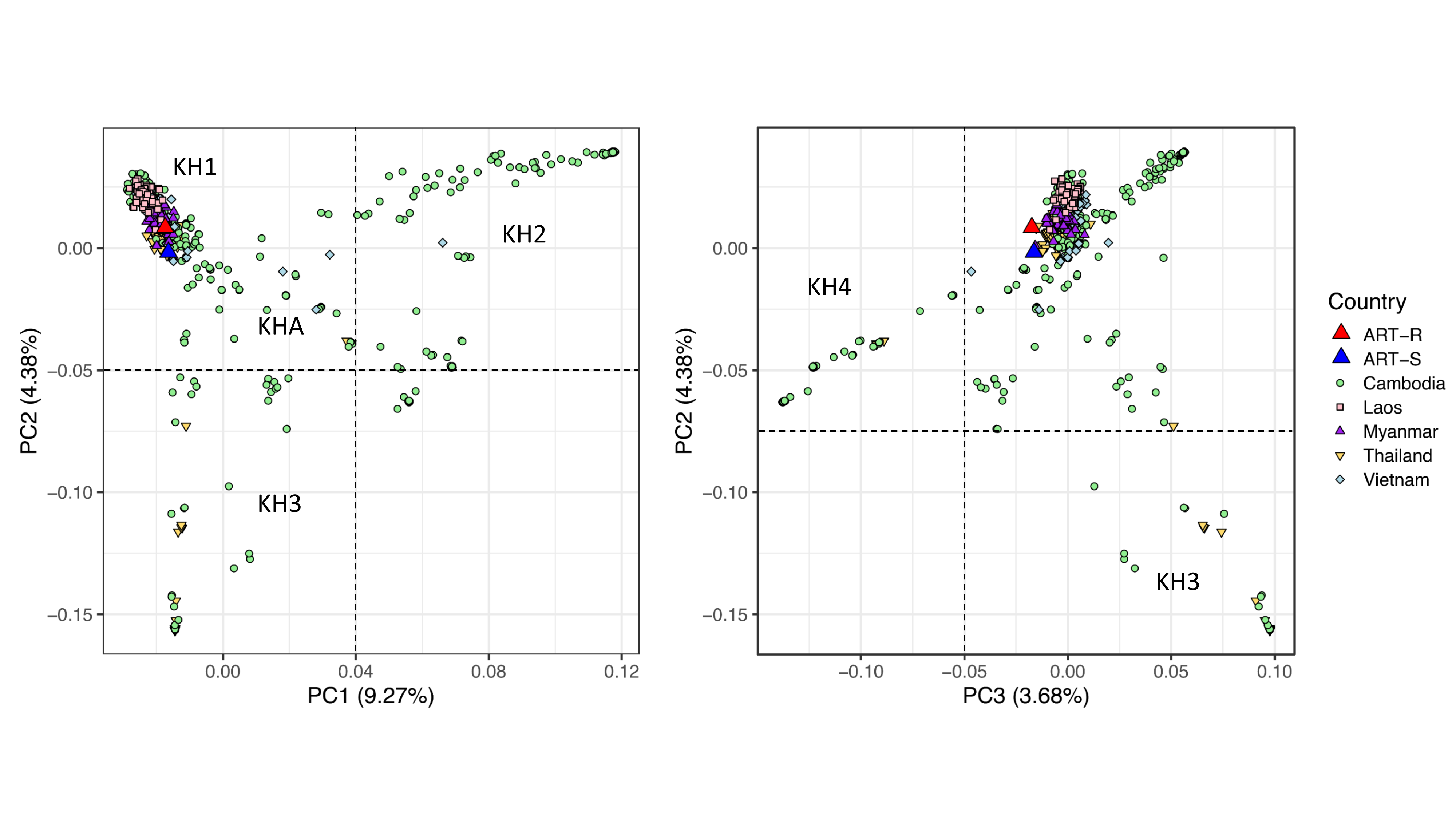

Supplement: S7 Fig — The genotype data was obtained from Sanger pf3k project (ftp://ngs.sanger.ac.uk/production/pf3k/release5/). The parent parasites from the cross analyzed in this study fall into KH1 group as defined by Miotto et al [35]. (TIF) [file pgen.1008453.s007.tif]

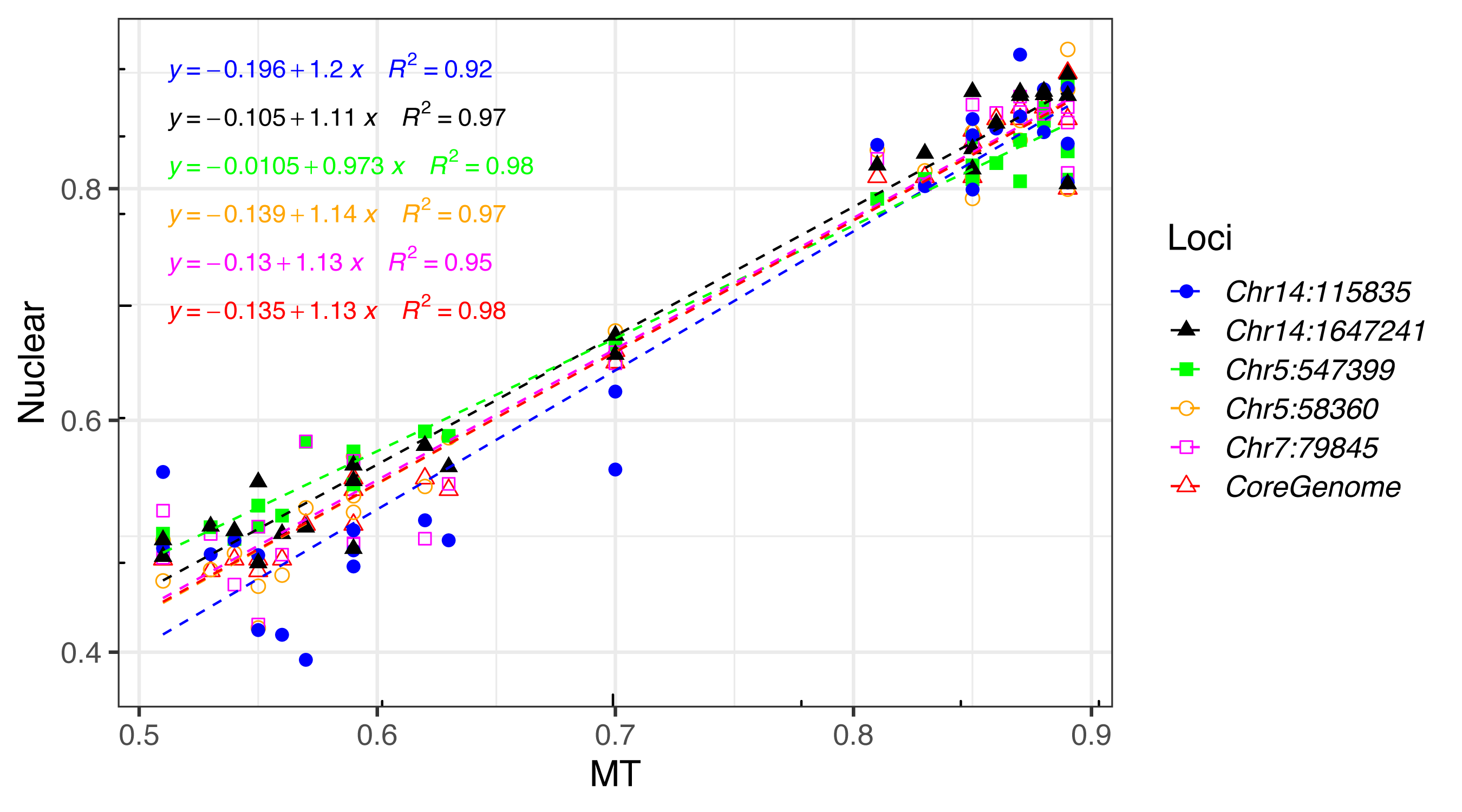

Supplement: S8 Fig — The strong correlations for different genome regions are consistent with selection against inbred parasites. (TIF) [file pgen.1008453.s008.tif]
