# Supplementary material for: Genetic mapping of fitness determinants across the malaria parasite Plasmodium falciparum life cycle
Source: PLoS Genet. 2019 Oct 14;15(10):e1008453. doi: 10.1371/journal.pgen.1008453 (PMC6821138; doi:10.1371/journal.pgen.1008453)
Supplement: S1 File — (DOCX) [file pgen.1008453.s015.docx]

**S1 File. Supplemental Methods and Materials**

**Parasites, mosquitoes and mice**

FRG NOD huHep mice [1] with human chimeric livers were purchased from Yecuris Corporation. Mice used in the study were supplemented with NTBC at 8 mg/L in their drinking water on arrival and maintained on this dose until euthanasia.

The *A. stephensi* mosquitoes used in this study were maintained at 27 °C and 75% humidity on a 12-h light/dark cycle. We followed MR4 protocols [2] for larval stage and adult stage rearing; larvae were fed with finely ground Tetramin fish food, and adults were fed with cotton balls soaked in a solution of 8% dextrose and 0.05% para-aminobenzoic acid in water.

*P. falciparum* blood stage cultures were maintained *in vitro* in standard cell culture media (RPMI-1640 with 25 mM HEPES and 2 mM l-glutamine supplemented with 50 μM hypoxanthine and 10% A+ human serum) [2]. An atmosphere of 5% CO_2_, 5% O_2_ and 90% N_2_ was used for growth, and infected *P. falciparum* red blood cells were subcultured into O+ erythrocytes.

We used two parasites isolated from hyper-parasitemic patients visited the Wang Pha clinic run by the Shoklo Malaria Research Unit (SMRU). *P. falciparum* NHP1337 (ART-R, C580Y mutant *kelch13*) and MKK2835 (ART-S, wild-type *kelch13*) isolates were grown in the laboratory, cloned by limiting dilution, and single parasite clones were used for these experiments.

**Genetic cross preparation**

To initial the cross, we set up asexual cultures of both parents by at 1% (mixed stages) parasitemia and 5% hematocrit. The cultures were maintained with daily medium changes for two weeks to enrich gametocytes. We then mixed gametocytes from each parent at equal ratio, and fed to adult female mosquitoes. This day was defined as day 0 for sample collecting (**Fig 1; Table 1**). Forty-eight midguts were dissected at each oocyst collection time point (day 4 and day 10). The prevalence of infection was analyzed at day 10. Salivary gland were separated to collect sporozoites at day 14 after infection. Sporozoites from 204 mosquitoes were mixed together for infection into the mouse and for isolation of genomic DNA.

Six days after sporozoite injection (day 20), we injected mice intravenously with 400 μL of packed O+ huRBCs. The intrave­nous injection was repeated the next day (day 21). Four hours after the second huRBC injection, mice were sacrificed and blood was removed by cardiac puncture in order to recover *P. falciparum*–infected huRBCs. The mouse liver was dissected, immediately frozen in liquid nitrogen and then stored at −80 °C. The blood was added to 10 mL complete medium (RPMI-1640 with 25 mM HEPES, 2 mM l-glutamine, and 50 μM hypoxanthine) and pelleted by centrifugation at 200g. We then removed the supernatant along with the buffy coat (containing white blood cells), and the red blood cells were washed three times with 10 mL complete medium, with pelleting and centrifu­gation as detailed above. After the third wash, an equal volume of packed O+ huRBCs (approximately 400 μL) was added, and the total RBC pellet was resuspended in complete medium to 2% hema­tocrit, and maintained in an atmosphere of 5% CO_2_, 5% O_2_ and 90% N_2_. Two days after culture, the parasites were split equally into two wells (repeat A and repeat B) of a standard six-well plate, and 50 μL of freshly packed huRBCs were added every 2 days to each well. Once parasitemia reached 4%, serial dilutions of parasites were carried out to maintain healthy cultures. The cultures were maintained for 30 days in total (day21-day50), and 50ul packed red blood cells (RBCs) were collected and frozen down every 2-4 days. We also set up gametocyte enrichment cultures from day 32 progeny population with daily medium changes but no fresh huRBCs. Samples were collected 8 days (day 40) and 16 days (day 48) later.

**Real-time quantitative PCR**

Real-time quantitative PCR (qPCR) reactions were performed in duplicate using the AB1 prism 7900HT Sequence Detection system (Applied Biosystems, Carlsbad, California, USA) as follows: 95 °C for 10 min, then 40 cycles of 95 °C for 15 s and 60 °C for 1 min. Duplicate reactions showing a difference in CT greater than one were rerun. We examined the melting curve (60–95 °C) at the end of each assay to verify the uniqueness of the PCR products generated. The reaction mixture consisted of 5 µL of SYBR Green MasterMix (Applied Biosystems, Carlsbad, California, USA), 0.3 µL of 10 µM primers (**S3 Table**) amplifying 67 bp of the *Pf_Tubilin* gene (PF3D7_1008700), 3.4 µL of sterile water and 1 µL of total DNA template. We plotted standard curves using seven dilutions at copies/µL from 2 × 10^1^ to 2 × 10^7^ with 10-fold interval of a purified *Pf_Tubilin* PCR product. The number of *Pf_Tubilin* copies in each sample was estimated according to the standard curve.

**Selective whole genome amplification**

sWGA reactions were performed following Oyola et al [3]. Each reaction (50 μl total volume) contained at least 0.2 × 10^6^ copies of *Plasmodium* DNA, 1× BSA (New England Biolabs), 1 mM dNTPs (New England Biolabs), 3.5 μM of each amplification primer [3], 1× Phi29 reaction buffer (New England Biolabs), and 30 units of Phi29 polymerase (New England Biolabs). The Phi29 polymerase used here is conducted at a single temperature and does not require denaturation/extension. We used a PCR machine (SimpliAmp, Applied Biosystems) programmed to run a “stepdown” protocol: 35 °C for 10 min, 34 °C for 10 min, 33 °C for 10 min, 32 °C for 10 min, 31 °C for 10 min, 30 °C for 3 h then heating at 65 °C for 10 min to inactivate the enzymes prior to cooling to 4 °C. Sample were cleaned with AMPure XP Beads (Beckman Coulter), at a 1:1 ratio. We quantified the amplified product using Qubit® dsDNA Broad Range (Thermo Fisher Scientific) to determine whether there was enough material for sequencing-minimum required is 50 ng.

**Ancestral/derived allelic state analysis**

Orthologous genes were identified using NCBI BLAST (<https://blast.ncbi.nlm.nih.gov/Blast.cgi>). We compared gene coding sequences from reference genomes of *P. falciparum* (Pf3D7), *P. reichenowi* (PrG01), *P. billcollinsi* (PbilG01), *P. blacklocki* (PblG01), *P. gaboni* (PgaG01) and *P. adleri* (PadlG01). A phylogenetic tree showing the evolution of *Laverania* ( Otto et al [4]) was used to infer the ancestry of *P. falciparum variants*. We performed codon-based multiple alignments using PRANK [5] with the -codon and + F options. Ancestral and derived allelic state was estimated by est-sfs [6] once the alignment was done.

# References

1. Azuma H, Paulk N, Ranade A, Dorrell C, Al-Dhalimy M, Ellis E, et al. Robust expansion of human hepatocytes in Fah−/−/Rag2−/−/Il2rg−/− mice. Nature biotechnology. 2007;25(8):903.

2. Moll K, Ljungström I, Perlmann H, Scherf A, Wahlgren M, Manassas V. Methods IN Malaria Research. 2008.

3. Oyola SO, Ariani CV, Hamilton WL, Kekre M, Amenga-Etego LN, Ghansah A, et al. Whole genome sequencing of Plasmodium falciparum from dried blood spots using selective whole genome amplification. Malaria journal. 2016;15(1):597.

4. Otto TD, Gilabert A, Crellen T, Böhme U, Arnathau C, Sanders M, et al. Genomes of all known members of a Plasmodium subgenus reveal paths to virulent human malaria. 2018;3(6):687.

5. Löytynoja A. Phylogeny-aware alignment with PRANK. Multiple sequence alignment methods: Springer; 2014. p. 155-70.

6. Keightley PD, Jackson BCJG. Inferring the probability of the derived vs. the ancestral allelic state at a polymorphic site. 2018;209(3):897-906.
